# Treatment planning system commissioning of the first clinical biology‐guided radiotherapy machine

**DOI:** 10.1002/acm2.13638

**Published:** 2022-05-29

**Authors:** Eric Simiele, Dante Capaldi, Dylan Breitkreutz, Bin Han, Timothy Yeung, John White, Daniel Zaks, Michael Owens, Srinath Maganti, Lei Xing, Murat Surucu, Nataliya Kovalchuk

**Affiliations:** ^1^ Department of Radiation Oncology Stanford University Stanford California USA; ^2^ RefleXion Medical, Inc. Hayward California USA

**Keywords:** BgRT, RefleXion, TPS commissioning

## Abstract

**Purpose:**

The RefleXion X1 is a novel radiotherapy machine designed for image‐guided radiotherapy (IGRT) and biology‐guided radiotherapy (BgRT). Its treatment planning system (TPS) generates IMRT and SBRT plans for a 6MV‐FFF beam delivered axially via 50 firing positions with the couch advancing every 2.1 mm. The purpose of this work is to report the TPS commissioning results for the first clinical installation of RefleXion™ X1.

**Methods:**

CT images of multiple phantoms were imported into the RefleXion TPS to evaluate the accuracy of data transfer, anatomical modeling, plan evaluation, and dose calculation. Comparisons were made between the X1, Eclipse™, and MIM™. Dosimetric parameters for open static fields were evaluated in water and heterogeneous slab phantoms. Representative clinical IMRT and SBRT cases were planned and verified with ion chamber, film, and ArcCHECK^@^ measurements. The agreement between TPS and measurements for various clinical plans was evaluated using Gamma analysis with a criterion of 3%/2 mm for ArcCHECK^@^ and film. End‐to‐end (E2E) testing was performed using anthropomorphic head and lung phantoms.

**Results:**

The average difference between the TPS‐reported and known HU values was −1.4 ± 6.0 HU. For static fields, the agreements between the TPS‐calculated and measured PDD_10_, crossline profiles, and inline profiles (FWHM) were within 1.5%, 1.3%, and 0.5 mm, respectively. Measured output factors agreed with the TPS within 1.3%. Measured and calculated dose for static fields in heterogeneous phantoms agreed within 2.5%. The ArcCHECK^@^ mean absolute Gamma passing rate was 96.4% ± 3.4% for TG 119 and TG 244 plans and 97.8% ± 3.6% for the 21 clinical plans. E2E film analysis showed 0.8 mm total targeting error for isocentric and 1.1 mm for off‐axis treatments.

**Conclusions:**

The TPS commissioning results of the RefleXion X1 TPS were within the tolerances specified by AAPM TG 53, MPPG 5.a, TG 119, and TG 148. A subset of the commissioning tests has been identified as baseline data for an ongoing QA program.

## 1 INTRODUCTION

Unlike treatment delivery errors, which are mostly random in nature,^1^ treatment planning system (TPS) errors are systematic and can be minimized through thorough commissioning followed by periodic quality assurance (QA). The American Association of Physicists in Medicine (AAPM) Task Group 53^2^ and AAPM Medical Physics Practice Guideline (MPPG) 5.a^3^ provide methodologies for TPS commissioning and QA. In general, commissioning and QA tests can be broken into several subsections including non‐dosimetric, dosimetric for static fields, and dosimetric for intensity‐modulated fields. Each subsection contains a variety of tests aimed at evaluating the TPS performance under clinically relevant scenarios (e.g., accuracy of contour generation, integrity of data import/export, output factor calculation accuracy)

The first biology‐guided radiotherapy (BgRT) system, RefleXion™ X1 (RefleXion Medical Inc, Hayward, CA, USA), was installed at our institution for clinical use for CT‐based IGRT and investigational use for BgRT. A detailed description of the relevant components on the X1 system is shown in Table 1. The X1 consists of a 6 MV flattening‐filter‐free (FFF) linear accelerator mounted on an 85 cm O‐ring gantry rotating at 60 rpm. Modulation is achieved via 50 firing gantry positions with 64 binary, pneumatically driven MLCs with a transition rate of 100 Hz. The width of a single leaf at isocenter (85 cm SAD) is 6.25 mm. Two sets of jaws, positioned above and below the MLCs, are used to set the maximum field extent in the patient superior‐inferior direction: either 1 or 2 cm at isocenter. The X1 is also equipped with fan‐beam kilovoltage computed tomography (kVCT), megavolt‐ age portal, and positron emission tomography (PET) imaging subsystems. Unlike helical Tomotherapy^@^ (Accuray Inc., Sunnyvale, CA), the treatment beam is delivered axially with the couch advancing 2.1 mm between beam stations in three treatment modes: IMRT, SBRT, and BgRT. The difference between these modes is the use of PET guidance (BgRT only) and the number of passes of the treated region (one pass for IMRT and four passes for SBRT and BgRT).

**TABLE 1:** A summary of the operating characteristics and components of the RefleXion™ X1 system

Hardware	
Source‐isocenter distance	85.0 cm
Bore diameter	85.0 cm
Gantry rotation speed	60 rpm

Linear accelerator	
Photon beam energy	6 MV flattening‐filter‐free
Dose rate	850 MU/min
Jaws	Two sets situated above and below the MLCs. Capable of 1 or 2 cm field widths in IEC‐Y
MLCs	64 binary, double‐focused, width of 6.25 mm at isocenter, maximum field width of 40 cm in IEC‐X
Firing positions	50 (i.e., every 7.2 degrees)
Beam delivery modes	IMRT (1 pass)/SBRT (4 passes)/BgRT (4 passes) Axial delivery in 2.1 mm table increments

TPS	
Optimization algorithm	Accelerated proximal gradient based on FISTA algorithm
Dose calculation algorithm	Collapsed cone convolution (CCC) superposition
Dose calculation grid size	2.1 mm
Tongue‐and‐groove	Included in beam model

kVCT	
Tube current	45‐400 mA
Tube voltage	120 kV
Imaging geometry	Fan beam
Acquisition mode	Helical with pitch = 0.22, 0.5, or 1.33
FOV	50 cm
Maximum scan length	95 cm
Slice thickness	1.25 mm
kVCT isocenter distance from treatment isocenter	38.6 cm

PET BgRT	
FOV (transverse)	50 cm
FOV (longitudinal)	5.0 cm
PET spatial resolution	4.0 × 4.0 × 2.1 mm^3^

Currently, BgRT is not FDA‐cleared for patient treatment, but is an active area of research. Therefore, the purpose of this study was to report the commissioning results of the X1 TPS with a specific focus on the IMRT and SBRT treatment modes. While the geometry of the X1 system is similar to other ring‐gantry setups (e.g., Tomotherapy^@^, Halcyon™), the commissioning process differs from these systems in that golden beam data is not provided with the X1 system. Therefore, the commissioning and QA tests proposed in AAPM TG 53, MPPG 5.a (TG 244), TG 106, TG 119, and TG 148 were utilized and adapted for the X1 system as described in the following sections ^2^, ^4^, ^5^, ^6^, ^7^: Version 1.0.19 of the RefleXion X1 TPS was installed at our institution in August 2020; however, since the original installation multiple upgrades and iterations were made to the TPS before the first patient treatment in May 2021. In this work, we present the commissioning report for the X1 TPS version 1.0.46.

## 2 METHODS

Prior to commissioning, acceptance tests were performed ensuring agreement within the vendor‐specified tolerances of the tested parameters. RefleXion X1 machine commissioning, Monte Carlo beam validation, and PET sub‐system commissioning are described in separate manuscripts.^8^, ^9^, ^10^ The scope of the TPS commissioning tests was designed based on our clinical environment and the clinical implementation of the X1 TPS. As the current TPS version does not offer fusion and contouring capability, its integration with Eclipse™ (v15.6, Varian Medical Systems, Palo Alto, CA, USA) and MIM™ (v7.1.2, MIM Software Inc, Cleveland, OH, USA) was evaluated. As recommended by AAPM TG 53, the commissioning tests were split into non‐dosimetric (Table 2) and dosimetric (Table 3) categories.

**TABLE 2:** A summary of the non‐dosimetric tests performed on the RefleXion X1 TPS

Image Input Tests	
1.1	DICOM CT and RTStruct data import
1.2	Integrity of text information transfer
1.3	Image geometry (number of pixels, slices, slice thickness)
1.4	Geometric location and orientation of scan
1.5	CT density curve
1.6	Transfer of CT, RTStruct, and RTDose between RefleXion, Eclipse, and MIM

Anatomical structure tests	
2.1	Structure attributes (name, type, color)
2.2	Relative electron density definition (overrides)
2.3	Couch structure and Body structure
2.4	Structures created from contours (Boolean operators)
2.5	Structures created from non‐axial contours
2.6	Integrity of imported structures from Eclipse and MIM

Image use and display tests	
3.1	Window/Level setting
3.2	Geometric accuracy of CT slices
3.3	ROI analysis (HU value within a ROI)
3.4	Tools in RefleXion tool panel
3.5	Position measurements

Plan setup tests	
4.1	Target localization position
4.2	Imaging protocols and extent
4.3	Safe zone (collision)

Dose display tests	
5.1	Static dose points
5.2	Isodose surfaces and dose cloud
5.3	DVH tests: dose min, max, mean, and DVH plotting

Plan and treatment delivery report tests	
6.1	Plan report: patient info, plan overview, treatment info
6.2	Plan report: DVH parameters, isodose display
6.3	Treatment delivery printout

The test number is shown in the first column and a short description is given in the second column.

**TABLE 3:** A summary of the dosimetric tests performed on the RefleXion X1 TPS

Dose profiles and PDDs in water	
7.1	PDDs: (1.25, 2.5, 5, 10, 20, 40) × 1 and 2 cm^2^; SSD = 85 cm
7.2	Profiles: (1.25, 2.5, 5, 10, 20, 40) × 1 and 2 cm^2^; depths = 1.5, 5, 10, 15, 20 cm; SSD = 85 cm

Point doses in water	
8.1	Output factors: (0.625, 1.25, 2.5, 3.75, 5, 7.5, 10, 15, 20, 30, 40) × 1 and 2 cm^2^; depth = 10 cm; SSD = 85 cm
8.2	Reference calibration condition: *D* (*d*_max_); 10 × 2 cm^2^; SSD = 85 cm

ArcCHECK® measurements	
9.1	Gantry = 0 : (1.25, 2.5, 5, 10, 20, 40) × 1 and 2 cm^2^; SAD = 85 cm
9.2	TG 244 plans: abdomen, thorax, lung, HN, prostate, and anal
9.3	TG 119 plans: prostate, HN, CShape easy, and CShape hard
9.4	Brain met SRS plans: target diameter = 1.5, 2, and 3 cm
9.5	Large target plan: prostate+nodes
9.6	Patient plans: 6 HN, 3 prostate, 3 lung, 3 anal, 3 GYN, and 3 lung SBRT plans

Plans in solid water phantom	
10.1	TG 119 plans: prostate, HN, CShape easy and hard
10.2	Small target plans: brain met SRS plans (target diameter = 1.5, 2, and 3 cm)
10.3	Large target plan: prostate+nodes

Solid Water phantom with heterogeneous slabs	
11.1	Gantry = 0:5 cm lung and 3 cm bone slabs

Off‐axis targets	
12.1	Brain SRS in CyberKnife head phantom; 5 cm lateral shift
12.2	SBRT single spherical target in Solid Water phantom; 5 cm lateral shift

End‐2‐end tests	
13.1	Lung SBRT plan on CIRS Dynamic Thorax Phantom—static delivery
13.2	Lung SBRT plan on CIRS Dynamic Thorax Phantom—dynamic delivery Amplitude = 20 mm, period = 6 s, direction = superior‐inferior
13.3	Brain SRS plan on CyberKnife head phantom

Beam interruption	
14.1	Interrupted plan and continuing delivery

Couch transmission	
15.1	Couch transmission

The test number is shown in the first column and a short description is given in the second column.

The input data for beam modeling consisted of percentage depth dose profiles (PDDs) along the beam central axis in water, relative beam profiles along the transverse (i.e., crossline) and longitudinal (i.e., inline) directions in air, and various MLC leaf‐opening combination profiles in air for tongue‐and‐groove modeling. The data was acquired using a Sun Nuclear Edge™ diode detector (Sun Nuclear Corporation, Melbourne, FL) in an IBA Blue Phantom Helix water tank (IBA dosimetry, GmbH, Germany) for field sizes ranging from 1.25 *×* 1 cm^2^ to 40 *×* 2 cm^2^. The X1 system was calibrated to deliver 1 cGy per one Monitor Unit (MU) for a 10 *×* 2 cm^2^ field at 85 cm SSD and 1.5 cm water‐equivalent depth following the methodology described by Mirzakhanian et al.^11^ In the current version of the TPS, the user does not have access to beam modeling workspace, thus, the vendor iterated the beam model based on the agreement between the calculated and measured verification data. The beam model optimization includes the following steps: open field fluence map modeling, MLC leaf modeling, energy spectrum fitting, absolute dose scale factor determination, validation data acquisition by the user, iteration of the steps above to minimize discrepancies between TPS and measurements. As the RefleXion X1 treatment is delivered with the small beamlets formed by a few MLC leaves and the narrow Y‐jaw openings which results in the lack of charge particle equilibrium, it is of paramount importance to ensure the accuracy of the small‐field dosimetry.^12^ The small tissue‐equivalent detectors were used for RefleXion X1 measurements to avoid volume averaging, fluence perturbation corrections, and dependence of detector response on the beam energy increase in phantom with decreasing field size.^13^, ^14^, ^15^, ^16^ The verification data consisted of profile scans and output factors measurements in water with the Edge detector and a tissue‐equivalent Exradin^@^ W2 scintillation detector (Standard Imaging Inc., Middleton, WI) for field sizes ranging from 0.625 *×* 1 cm^2^ to 40 *×* 2 cm^2^.

### 2.1 Non‐dosimetric tests

#### 2.1.1 Image input and anatomical structure tests

Multiple CT scans acquired with various CT scanners were imported into the X1 TPS to evaluate the integrity of information transfer. Items evaluated included patient name, patient ID, date of birth, CT scanner Hounsfield Unit (HU) calibration curve assignment, and patient orientation. Image geometry integrity was evaluated by comparing the reported number of pixels, pixel size, number of slices, and slice thickness in the X1 TPS to the values reported from the CT scanner used to acquire the image.

The HU versus mass density curve definition in the X1 TPS requires a vacuum point where the mass density is 0.0 g/cc. This vacuum point is needed because the X1 TPS automatically performs couch replacement in the structure set where the added RefleXion couch structure density is below the normal range of material densities used in a standard HU to mass density curve. CT scans of the CIRS Electron Density phantom (Computerized Imaging Reference Systems, Inc., Norfolk, VA) were acquired and imported into the RefleXion TPS and the reported HU values were verified against measured HU values on the CT scanners.

The integrity of CT, RT structure, and RT dose file transfer between RefleXion, MIM, and Eclipse was tested as it is required by our clinical workflow for the current implementation of the X1 system. The AAPM TG 244^7^ head‐and‐neck data set was used for comparison in structure volumes between RefleXion, Eclipse, and MIM. Currently, the X1 TPS does not allow density overrides using structures. Therefore, density overrides for these cases are achieved by modifying HU values of the CT scan in MIM. The integrity of density overrides using this method was evaluated on the X1 TPS by overriding a region of air in a CT scan to a HU of 0.0 (i.e., water).

While no contouring tools are available in the current version of the X1 TPS, various Boolean operations (e.g., union, intersection, subtraction, etc.) on existing structures can be performed. However, these Boolean volumes cannot be visualized in the X1 TPS. The accuracy of each available Boolean operator was tested on a spherical contour from one of the AAPM TG 244 data sets^7^ where the same operation was performed in both the X1 and Eclipse TPS and the reported volumes were compared.

#### 2.1.2 Image use and display tests

The imported Electron Density phantom CT images from the previous section were used to evaluate the accuracy of image display in the X1 TPS. In addition, the available image analysis tools were also tested for functionality and accuracy. The probe tool was used to read the HU at the center of each material insert in the CIRS phantom and the constancy of the reported HU to changes in the window‐level setting was evaluated. The geometrical accuracy of displayed images was tested by measuring the dimensions of an object of known size using the measuring tool.

A CT scan of the AAPM TG 119^5^ Solid Water™ (Gammex Inc., Middleton, WI) phantom setup was acquired, imported in the X1 TPS, and the length, width, and height of the phantom were measured and compared to the known dimensions (length = 300.0 mm, width = 300.0 mm, and height = 160.0 mm). The reported absolute DICOM positions of two points in the TG 119 phantom image were compared between the X1 TPS and Eclipse. This test was also used to evaluate the target localization position in the X1 TPS as described in the next section.

#### 2.1.3 Plan setup tests

In the X1 TPS, the treatment isocenter can be placed manually using the probe tool or the user can select a structure in which to place the isocenter where the isocenter is assigned to the centroid of that structure. The accuracy of auto‐assigning the isocenter to a structure in the X1 TPS was tested using the CT image of the TG 119 phantom. The reported DICOM position in the X1 TPS was compared to the reported position in the Eclipse TPS for a marker‐type structure and the contoured ionization chamber structure.

To minimize the possibility of a collision between the patient and the bore, the proposed isocenter placement in the X1 TPS is compared against a hard‐coded collision zone. The accuracy of this collision zone was compared to the measured collision zone on the RefleXion system. After selecting the treatment isocenter, the planner must specify the scan extent and protocol to be used for daily kVCT of the patient. The scan extent functionality was tested to ensure the specified scan range and direction were accurately displayed on the image and the specified range could not exceed the length of the CT scan.

#### 2.1.4 Dose display tests

The accuracy of the displayed dose was tested by comparing point doses, isodose surfaces, and DVH curves between the X1 TPS, Eclipse, and MIM. A mock plan was generated in the X1 TPS on a previously imported patient CT scan and the reported dose in a high‐dose, low‐ gradient region was recorded along with its 3D DICOM coordinates. The RT dose file was exported to Eclipse and the reported dose at the same DICOM coordinates was compared to the X1‐reported dose. The accuracy of reported isodose surfaces was evaluated using the probe tool to ensure point doses on each isodose surface matched the reported dose level. A mock head‐and‐neck plan was generated on a previously imported patient CT scan in the X1 TPS. The RT dose file from the plan was exported to MIM and Eclipse and the reported structure minimum, maximum, and mean doses were compared between the three systems for 17 structures of varying size and shape.

#### 2.1.5 Plan report and treatment delivery tests

Using the mock head‐and‐neck plan generated in the previous section, the plan report was printed and checked to ensure the printed information matched the displayed information in the TPS. In addition, the plan report was evaluated to ensure it contained the relevant information for a given patient's treatment plan (i.e., patient name, date, prescription, plan type, dose, TPS version, beam model version, total MU, DVH curves for all structures). Finally, the mock plan was treatment approved in the TPS, delivered on the X1 system, and the treatment report was printed, compared to the plan report and the information displayed in the TPS, and then inspected for accuracy of the parameters that were delivered during treatment (e.g., delivered MU).

### 2.2 Dosimetric tests

The accuracy of the generated beam model (Section 2) was evaluated based on comparisons with measured static field point doses and profiles, AAPM TG 119, TG 244, and 21 representative clinical plan QA measurements, and end‐to‐end (E2E) testing. Criteria for passing was set based on TG 53,^2^ MPPG 5.a^3^ (TG 244), and TG 148^6^ depending on the test and region of the beam tested.

#### 2.2.1 Static field analysis

Measured PDDs, crossline and inline profiles (1 mm resolution), and output factors in static open fields were compared to the calculated dose in water from the X1 TPS. Field sizes ranging from 1.25 cm to 40 cm in the crossline direction for both jaw openings (1 cm and 2 cm) were tested for 85 cm SSD. All profile measurements were performed in the IBA Blue Phantom Helix water tank using a Sun Nuclear Edge diode detector. Output factor measurements were performed at 10 cm depth in water using a combination of the Edge diode detector and the Exradin W2 scintillation detector. A reference calibration check was performed in the X1 TPS to ensure correct assignment of the dose per delivered MU in the system. For a static 10 *×* 2 cm^2^ field incident on a water phantom at 85 cm SSD, 500 MU was specified in the system, and the point dose at *d*_max_ was recorded and compared to the expected value of 500 cGy.

A 1D Gamma analysis^17^ was used to compare the measured and calculated PDDs and crossline profiles with Gamma criteria of 1%/1 mm. In addition, PDD_10_ and the mean dose difference in 80% of the nominal field width were also calculated for the PDDs and crossline profiles, respectively. The calculated FWHM of the inline profiles was compared between the measured and calculated data. In the current version of the X1 TPS, the dose grid resolution is fixed at 2.1 mm and cannot be adjusted. With the assistance of the manufacturer, all static plans on Water Phantom were generated for the default dose grid resolution as well as a resolution of 1 mm. Due to the limited dose evaluation and data extraction tools in the current X1 TPS version, the RT Dose files from the profile plans were exported and data analysis was performed in both Eclipse or MATLAB^@^ (The MathWorks, Inc., Natick, MA). If MATLAB was used, scripts were written to extract the relevant data directly from the RT dose file. For the dose files that were imported into Eclipse, an Eclipse API script was written to expedite data extraction from the dose distribution.

#### 2.2.2 Static fields with heterogeneities

Dose calculation accuracy in the presence of heterogeneities was tested by creating static field plans irradiating a 3 cm bone and 5 cm lung slabs sandwiched between two 30 *×* 30 *×* 5 cm^3^ Solid Water slabs. Point doses along the beam central axis were extracted 1 cm upstream and downstream from the heterogeneity insert and were compared to the measured dose in a Solid Water phantom with lung or bone heterogeneity slabs using an Exradin^@^ A14SL ionization chamber (Standard Imaging Inc., Middleton, WI). Plans were generated for field sizes of 10 *×* 1 cm^2^, 40 *×* 1 cm^2^, 10 *×* 2 cm^2^, and 40 *×* 2 cm^2^.

#### 2.2.3 Beam interruption and couch transmission

Prior to testing the dosimetric accuracy of delivered clinical plans, the impact of beam interruption on plan delivery was tested. Specifically, the abilities of the X1 treatment delivery computer and the X1 TPS to handle beam or plan interruptions and record the fraction of the total MU that were delivered before beam interruption. In addition, the system was also tested to ensure it could resume delivery after beam interruption. The accuracy of couch transmission modeling in the X1 TPS was tested by calculating and delivering plans with static 10 *×* 2 cm^2^ fields with gantry angles equal to 0, 180, and 187 onto a Solid Water phantom with a PTW TN31014 PinPoint^@^ ionization chamber (Freiburg, Germany) in the center of the phantom (85 cm SAD). The 187 gantry angle was included for testing the couch transmission as the X1 system contains a low‐activity PET source located at a gantry angle of 180 used for machine performance checks.

#### 2.2.4 Representative clinical plans

Clinical plans were generated for various treatment sites including head‐and‐neck, prostate, and lung, anal, Cshape IMRT and thorax SBRT following the AAPM TG 119 and TG 244 methodologies. Additional 21 patient plans were also created for various treatment sites (six head‐and‐neck, three prostate, three lung, three anal, three GYN, and three lung SBRT). All plans were delivered on the Sun Nuclear ArcCHECK^@^ (Melbourne, FL) and Gamma criteria of 3%/2 mm^18^ were used to evaluate the agreement between the measured and calculated dose distributions.

Prior to plan delivery, the ArcCHECK^@^ was calibrated on a Varian Truebeam™ linear accelerator (Varian Medical Systems, Palo Alto, CA) with 6 MV flat beam following the manufacturer‐recommended relative dosimetry calibration procedure. Absolute dose calibration of the ArcCHECK^@^ was performed on the RefleXion X1 system for a 10 *×* 2 cm^2^ field size. The accuracy of the ArcCHECK^@^ calibrations were tested by delivering static fields at multiple gantry angles and field sizes and comparing the measured central axis dose to the TPS‐calculated dose and performing Gamma analysis with criteria of 3%/2 mm.

A subset of the above plans were also delivered on a Solid Water phantom with a PTW PinPoint ionization chamber and Gafchromic™ EBT3 film (Ashland Inc., Bridgewater, NJ) (i.e., TG 119 measurement setup). The PinPoint‐measured dose was compared to the TPS‐reported mean dose to the chamber air volume. In addition to the TG 119 plans, four small spherical target plans (diameters ranging from 1–3 cm) and one large target plan were delivered on Solid Water. The small target plans were designed to represent single brain metastasis cases whereas the large target plan was representative of a prostate+nodes treatment.

A calibration curve was established for the EBT3 film in the dose range of 0–800 cGy on a Truebeam linear accelerator for a photon beam energy of 6 MV FFF. Gamma criteria of 3%/2 mm were used to evaluate the planar dose agreement between the film and TPS. All films were scanned on an EPSON™ Expression 10000 XL flatbed scanner (Los Alamitos, CA) using 48‐bit color with a resolution of 72 dpi for at least 24 h after radiation delivery. All film analysis (i.e., conversion from netOD to dose, alignment to TPS‐calculated dose, Gamma analysis, etc.) was performed using FilmQA Pro™ (Ashland, Inc., Bridgewater, NJ).

#### 2.2.5 Special treatment cases

In addition to testing common treatment sites described in the previous section, the performance of the X1 TPS to special treatment cases was also evaluated including targeting accuracy, delivery accuracy to off‐axis targets, and the impact of motion on the delivered dose distribution. Targeting accuracy was evaluated using the CyberKnife head phantom, which contains a hidden spherical target with orthogonal film inserts through the target. The objective is to accurately deliver a spherical dose distribution whose center (as determined from the orthogonal film measurements) coincides with the centroid of the hidden target. Any deviation between the centers of the dose distribution and the target indicate a systematic offset in targeting accuracy.

The Cyberknife head phantom was scanned in the head‐first‐supine position on a Siemens Biograph™ CT scanner (Siemens Medical Solutions, Inc., Malvern, PA) with a slice thickness of 1.2 mm. A treatment plan (prescription of 600 cGy per fraction in 10 fractions) was created on the X1 TPS with the isocenter located in the center of the 3‐cm‐diameter target in the Ball Cube II insert. The plan was delivered on the X1 system and the EBT3 film was scanned 24 h post‐irradiation using the equipment and parameters described in the previous section except the film was scanned using 16‐bit grayscale at 300 dpi. The E2E film analysis software provided by Accuray was used to analyze the scanned film.^19^ In addition to performing the targeting test with the phantom positioned at machine isocenter, the test was repeated with the phantom shifted laterally by 5 cm. A quantitative measure of treating off‐axis targets was obtained by re‐planning and delivering the single brain metastasis case (3‐cm‐diameter target) with the phantom shifted laterally by 5 cm.

The impact of motion on dose delivery accuracy was evaluated using the CIRS™ Dynamic Thorax Phantom with the SBRT 3 cm target insert (Computerized Imaging Reference Systems, Inc., Norfolk, VA). Two treatment plans were created for the CIRS phantom: one with no motion (i.e., static) and one with sinusoidal motion (amplitude = 20 mm, period = 6 s, motion direction = superior‐inferior). Static and 4DCT scans of the CIRS phantom were acquired without and with motion, respectively, on a Siemens Biograph CT and were imported into Eclipse. For the motion scan, a motion‐inclusive ITV was contoured and an ITV‐PTV expansion of 5 mm was applied to generate the PTV. For the static case, the contoured target was considered to be the PTV (i.e., no expansion). The average CT scan of all breathing phases was used for treatment planning in the X1 TPS. The prescription for these plans was 2500 cGy in five fractions (500 cGy per fraction), PTV V100%*≥*95%, and ITV *D*_max_ *<* 130%. The generated plans were delivered on the CIRS phantom with and without motion where the dose distribution in the sagittal plane through the center of the target was measured with EBT3 film. The film was analyzed and compared to the calculated dose distribution using the methods described in the previous section.

## 3 RESULTS

### 3.1 Non‐dosimetric tests

#### 3.1.1 Image input and anatomical structure tests

All DICOM header information was imported successfully into the X1 TPS with minor exceptions. In the current X1 TPS version, the user does not have access to TPS administration to configure the CT density curves. The user provides the information on the CT scanners with the corresponding curves to the vendor. The vendor then hard‐codes the CT density curves for each scanner. Thus, if a CT scan from an outside institution or the scanner information is not present in the DICOM header, the X1 TPS will throw a fault and not allow import of the data, which is an important consideration for TG‐244 data sets. For the imported CT scans, all header information was correctly identified and displayed in the X1 TPS, including scanner ID, scan orientation, number of slices, slice thickness, and number of pixels.

The maximum difference between the HU values measured on the X1 TPS and our Siemens Biograph CT scanner was 15.7 HU for the dense bone material (0.8 g/cc) in the CIRS Electron Density phantom. The average HU difference over all material inserts was 1.4 ± 6.0 HU. The CT, RT structure, and RT dose DICOM files were transferred successfully and accurately for five patients between the X1 TPS, MIM, and Eclipse with one exception: Eclipse could not directly import the exported RT dose from the X1 TPS. This was caused by a missing type 1 DICOM tag (ReferencedRTPlanSequence) in the RT dose file that Eclipse requires for import (MIM does not require this tag). To overcome this limitation, an Eclipse API script was developed to correct the DICOM header in the RT dose file to allow for direct import of the exported dose file into Eclipse.

The mean and maximum volume differences between the X1 TPS and Eclipse for the TG 244 head‐and‐neck structure set were 2.4 and 20.2 cc, respectively. The mean and max volume differences between the X1 TPS and MIM were 0.2 and 2.9 cc, respectively. Derived CT scans created in MIM to generate bolus were successfully imported and displayed in the X1 TPS. The maximum difference in volume between the Boolean volumes generated in Eclipse and the X1 TPS was 7.6 cc where the majority of differences were less than 1.0 cc. The volume difference between Eclipse and the X1 TPS for a bladder structure created from non‐axial contours was 0.3 cc.

#### 3.1.2 Image use and display tests

The available image analysis tools in the X1 TPS were all functional. There were no changes in the measured HU of multiple material inserts in the CIRS Electron Density phantom to varying window‐level setting. The maximum difference between the TPS‐measured dimensions of the TG 119 Solid Water and the known dimensions was 0.6 mm. The magnitude of the difference in position between Eclipse and the X1 TPS for the centroid of the chamber volume in the TG 119 phantom was 0.2 mm.

#### 3.1.3 Plan setup tests

The current version of the X1 TPS does not permit definition of a user reference location in a CT scan (typically the plane of the BBs defined at simulation) and does not enable visualization of the selected plan isocenter. Therefore, to clearly and unambiguously define the isocenter position for all RefleXion treatment plans, a marker structure was inserted into the structure set at the desired isocenter location in Eclipse. The magnitude of the difference in position between Eclipse and the X1 TPS for the marker placed in the TG 119 phantom was 0.1 mm.

Upon comparison between the measured and the hard‐coded TPS collision zones, deviations were observed where the TPS was overestimating collision in some areas (i.e., TPS collision zone was within measured collision zone), but underestimating collision for large lateral shifts and small couch vertical (i.e., measured collision zone was within TPS collision zone). In these underestimated collision areas, there is a potential for false negative reporting of collisions. Therefore, the overlap of the two collision zones was used to create a chart for dosimetrists to show the acceptable positions for isocenter placement (see Appendix). In addition, an Eclipse API script was developed to automatically check the marker (i.e., isocenter) placement against the determined collision zone before exporting the structure set to the X1 TPS. The kVCT scan extent specification tool in the X1 TPS was found to be accurate in magnitude and direction.

#### 3.1.4 Dose display tests

The static point dose measured in the high‐dose, low‐gradient region on the X1 TPS agreed with the Eclipse‐reported dose (difference = 0.0 cGy), indicating proper alignment of the imported RT Dose grid on the CT image in Eclipse, which is critical for accurate plan quality evaluation. The static point doses measured with the probe tool agreed with the displayed isodose surfaces. As the X1 TPS can display calculated dose as isodose lines or a colorwash or both, the display accuracy of the dose cloud (colorwash) was evaluated using the thresholding of the dose cloud and comparing it to isodose. The isodose surfaces were also converted to contours in Eclipse and compared using thresholding in RefleXion X1 TPS.

A summary of the differences in the reported DVH doses between the three systems (X1, Eclipse, and MIM) are shown in Table 4. On average, the X1‐reported doses agreed better with MIM‐reported doses as compared to Eclipse for the mock head‐and‐neck plan. The maximum discrepancy for all structure DVH doses was 506.9 cGy for the lips *D*_min_ between Eclipse and the X1 TPS. While the range of dose differences was large (see the Min(∆D) and Max(∆D) rows of Table 4), the mean doses were more consistent between the three systems with a maximum mean difference of 34.9 cGy (between X1 and Eclipse).

**TABLE 4:** The minimum, maximum, mean, and standard deviation of the DVH *D*_min_, *D*_max_, and *D*_mean_ reported from X1, Eclipse, and MIM for the same head‐and‐neck plan

	DVH *D*_min_, cGy		DVH *D*_max_, cGy		DVH *D*_mean_, cGy	
	X1−Eclipse	X1−MIM	X1−Eclipse	X1−MIM	X1−Eclipse	X1−MIM
Min (∆*D*)	−0.3	0.0	−171.6	−68.0	−15.7	−15.0
Max (∆*D*)	506.9	171.0	42.0	0.0	34.9	5.0
Mean (∆*D*)	31.3	13.5	−34.8	−23.4	6.7	−0.8
*σ*(∆*D*)	122.6	41.0	55.3	26.5	16.0	5.5

*Note* ∆*D* represents the dose difference between two systems for a given DVH metric (i.e., *D*_min_, *D*_max_, or *D*_mean_).

#### 3.1.5 Plan report and treatment delivery tests

All information relevant to the treatment plan (i.e., patient name, MRN, treatment technique, total MU, treatment time, etc.) was contained in the plan report printout except the CT scanner used to acquire the image, the selected imaging protocol on the X1 system, and selected imaging extent. A DVH plot of all structures and each structure volume, minimum dose, maximum dose, and mean dose are also printed as part of the plan report. All printed DVH curves and parameters agreed with the values displayed in the X1 TPS.

### 3.2 Dosimetric tests

#### 3.2.1 Static field analysis

**FIGURE 1:**
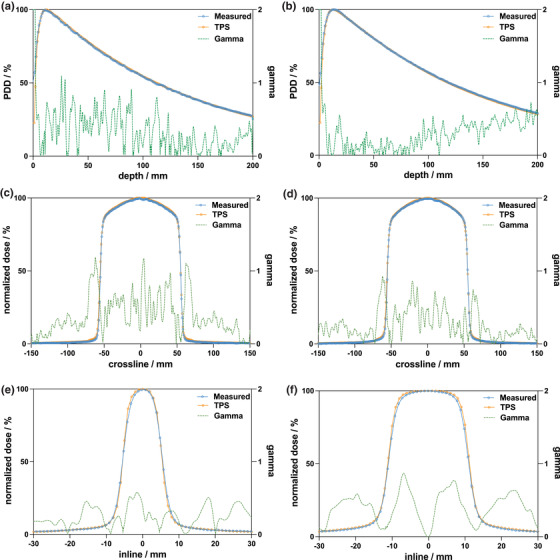
Measured and TPS‐calculated PDDs for the (a) 10 *×* 1 cm^2^ and (b) 10 *×* 2 cm^2^ field sizes. Measured and calculated crossline (orthogonal to direction of couch motion) profiles for (c) 10*×*1 cm^2^ and (d) 10 *×* 2 cm^2^ fields. Measured and calculated inline (parallel to direction of couch motion) profiles for (e) 10 *×* 1 cm^2^ and (f) 10 *×* 2 cm^2^ fields. All measurements and calculations were performed in a homogeneous water phantom at an SSD of 85 cm. Measurements were performed with the Sun Nuclear Edge diode in the IBA Blue Phantom Helix water tank. Gamma criteria of 1%/1 mm were used to compute the Gamma index

Representative measured and TPS‐calculated PDDs and profiles for the 10 *×* 1 cm^2^ (a, c, e) and 10 *×* 2 cm^2^ (b, d, f) fields are shown in Figure 1. The agreement between the measured and calculated data was better when the 1 mm dose grid was used as compared to the 2 mm dose grid. Specifically, the mean difference between the measured and calculated data for the crossline and inline profiles decreased and consequently, the gamma passing rate increased, due to the increased spatial resolution in the TPS dose distribution. Although the 1 mm dose grid is not currently available for clinical use, the comparisons between the measured and calculated profiles in this work are only described for the 1 mm dose grid as this data represents the highest accuracy achievable with the X1 TPS.

The maximum and mean percent differences between the measured and calculated PDD_10_ values were 1.5% (1*×*40 cm^2^) and 0.6%, respectively. The Gamma passing rates for depths greater than *d*_max_ (1.5 cm) were greater than 85.8% for all fields except the 2*×*1.25 cm^2^ field, which had a passing rate of 77.1%. Depths shallower than *d*_max_ were excluded from Gamma analysis in this work as the agreement between measurement and calculation is known to be poor in the build‐up region.^2^ The mean Gamma passing rate was 94.9% for all fields. The maximum mean difference in 80% of the crossline profile width was 2.6% whereas the average difference was 0.3% for all fields and depths investigated. For crossline field widths greater than 12.5 mm, the max and average mean differences decreased to 1.3% and 0.1%, respectively.

**FIGURE 2:**
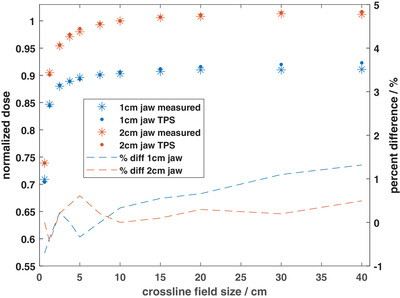
Measured and TPS‐calculated output factors for 1 cm and 2 cm jaw sizes. All measured and calculated doses were normalized to their respective doses at a field size of 10 *×* 2 cm^2^. Measurements were performed using a combination of a Sun Nuclear Edge diode detector and a Standard Imaging W2 plastic scintillation detector at 10 cm depth in water. TPS calculations were performed with a 1 mm^3^ dose grid. The output factor data correspond to the left *y*‐axis while the percent difference data correspond to the right *y*‐axis

The maximum and mean differences in the FWHM between the measured and calculated profiles were 0.5 and 0.0 mm, respectively. The FWHM for the calculated profiles was observed to consistently underestimate and overestimate the measured FWHM for all crossline fields and depths for the 1 and 2 cm jaw fields, respectively. The measured and TPS‐calculated output factors are shown in Figure 2 for both jaw field widths. The maximum discrepancy for all field sizes was 1.3%, whereas the mean difference was 0.2%. The TPS‐reported dose at *d*_max_ under reference conditions for 500 MU was 501 cGy (difference of 0.1%).

#### 3.2.2 Static fields with heterogeneities

The maximum discrepancy observed for the bone heterogeneity case was 2.5% at 9 cm depth for the 10 *×* 2 cm^2^ field. The discrepancy between the measured and calculated dose was, on average, greater downstream of the bone slab at 9 cm depth (mean difference = 2.1%) compared to upstream of the bone slab at 4 cm (mean difference = 0.7%). The mean overall difference for the bone heterogeneity was 0.7%. The maximum and mean difference for the lung heterogeneity was 2.1% and 0.6%, respectively. Similar to the bone slab, the agreement between the measured and calculated dose was greater upstream of the heterogeneity.

#### 3.2.3 Beam interruption and couch transmission

The X1 system and associated TPS were able to handle beam interruptions accurately. When a beam interruption occurred, the partial treatment was recorded in the X1 TPS as a partial delivery. The X1 system was able to accurately resume treatment at the interruption point and deliver the *planned MU − delivered measured MU* whether the patient plan remained open or was closed then re‐opened.

Due to the presence of the PET source located at gantry = 180*^◦^*, the measured dose difference between the gantry = 0*^◦^* and gantry = 180*^◦^* values was 12.2% with the TPS accurately modeling gantry = 180*^◦^* beam within 0.02%. At the gantry angles bypassing the PET source, that is, 187*^◦^*, the measured transmission and TPS‐calculated transmission agreed within 0.5% (3.5% vs 4.0%).

#### 3.2.4 Representative clinical plans

A total of 34 treatment plans were delivered to the ArcCHECK^@^ phantom, including TG 119 (8), TG 244 (5), and representative clinical cases (21). A summary of the measurement results for the TG 119 plans, both on Solid Water and ArcCHECK^@^, are shown in Table 5.

**TABLE 5:** A summary of the measurement results for the TG 119, single brain metastasis, and pelvis IMRT plans in Solid Water (film and PinPoint ionization chamber)

	PinPoint chamber		Film		
Plan	Location	%diff*	Location	γ 3%/2 mm	ArcCHECK® γ 3%/2 mm
TG119 Prostate IMRT	High dose/low gradient	0.4%	2.5 cm POST/low dose	99.8%	100.0%
TG119 HN IMRT	High dose/low gradient	−0.4%	4 cm POST/low dose	95.6%	99.3%
TG119 CShape Easy IMRT	Low dose/high gradient	−2.6%	2 cm ANT/high dose	94.6%	98.1%
TG119 CShape Hard IMRT	Low dose/high gradient	−2.4%	2 cm ANT/high dose	97.2%	98.1%
Pelvis IMRT	High dose/low gradient	2.2%	2.5 cm POST/low dose	95.3%	97.7%
Brain Met (d = 1.5 cm) SBRT	High dose/high gradient	7.9%	iso/high dose	100%	93.4%
Brain Met (d = 2.0 cm) SBRT	High dose/low gradient	0.6%	iso/high dose	96.9%	93.0%
Brain Met (d = 3.0 cm) SBRT	High dose/low gradient	0.0%	iso/high dose	96.2%	93.7%
Mean		0.7%		97.0%	96.7%
*σ*		3.1%		1.9%	2.8%

The same plans were also recalculated and delivered to the Sun Nuclear ArcCHECK®. The film and ArcCHECK® measured doses were compared to the planned doses using a Gamma analysis with criteria of 3%/2 mm. The percent difference (relative to the prescription dose) was calculated between the PinPoint‐measured dose and the TPS‐reported mean dose to the sensitive air volume of the chamber.

Agreement within 2.2% (relative to the prescription dose) was observed between the PinPoint‐measured and TPS‐calculated dose to the chamber volume when the chamber was positioned in a high‐dose, low‐gradient region. The agreement decreased to 2.6% and 7.9% when the chamber was positioned in low‐dose, high‐gradient and high‐dose, high‐gradient regions, respectively. The mean difference between the chamber‐measured and TPS‐calculated dose for all plans tested was 0.7%. The minimum film Gamma passing rate was 94.6% and the mean passing rate was 97.0% for Gamma criteria of 3%/2 mm. Similar results were observed for the ArcCHECK^@^ measurements using Gamma criteria of 3%/2 mm (minimum passing rate of 93.0% and mean passing rate of 96.7%). The minimum and mean passing rates for the clinical and TG 244 cases (non‐TG 119) delivered to the ArcCHECK^@^ were 87.4% and 97.5%, respectively.

**FIGURE 3:**
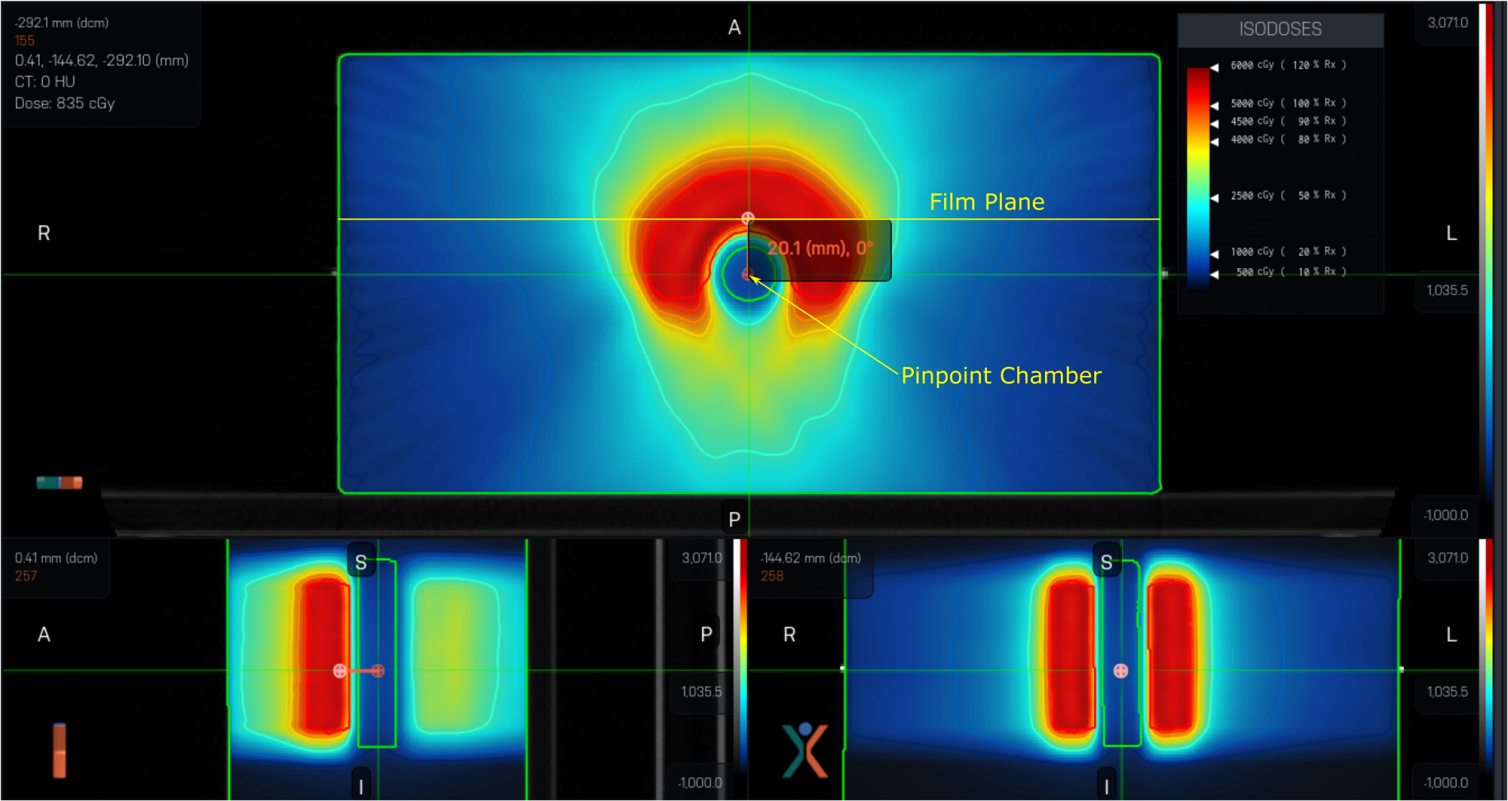
Dose distribution for the TG 119 C‐shape hard plan in the X1 TPS. The film measurement plane and the location of the PinPoint ionization chamber are indicated in the axial view. The prescription dose for this plan was 5000 cGy in 25 fractions (200 cGy per fraction)

**FIGURE 4:**
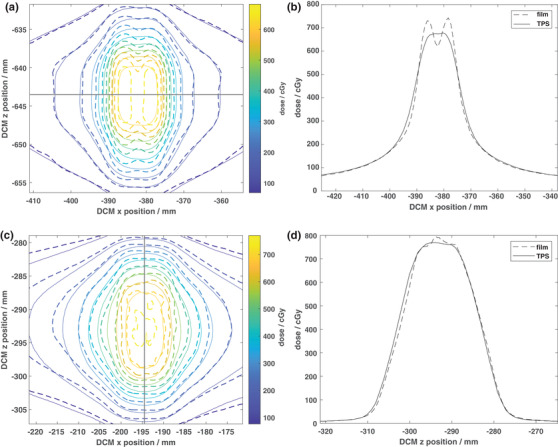
EBT3 film‐measured (dashed lines) and TPS‐calculated (solid lines) isodose distributions for the 600 cGy per fraction single brain metastasis plans for target diameters of (a) 1 cm and (c) 1.5 cm delivered on Solid Water. The solid black lines indicate the locations of 1D profiles through the dose distributions as shown in (b) and (d). As seen in b), the RefleXion X1 TPS reached the target size limit to accurately model the dose distribution for the 1 cm diameter target where only two MLC leaves participate in dose modulation. This limitation is resolved by increasing the target diameter by 5 mm as shown in (d)

A screenshot of the dose distribution, including the location of the film and PinPoint chamber, in the X1 TPS for the TG 119 CShape hard plan is shown in Figure 3. Film (dashed lines) and TPS (solid lines) isodose distributions for the single brain metastasis 1 cm and 1.5 cm cases are shown in Figures 4a and 4c, respectively. Profiles through the center of the target are shown for both cases in Figure 4b,d. Good agreement can be seen between the measured and calculated dose distributions for the 1.5 cm case in Figure 4c,d. However, significant discrepancies were apparent for the 1 cm‐diameter case (Figure 4a,b). Multiple sets of film measurements were performed for the 1 cm case, all producing similar results to those shown in Figure 4a,b. The ion chamber measurement in the center of the target was 18.5% lower than the TPS‐calculated dose.

#### 3.2.5 Special treatment cases

**FIGURE 5:**
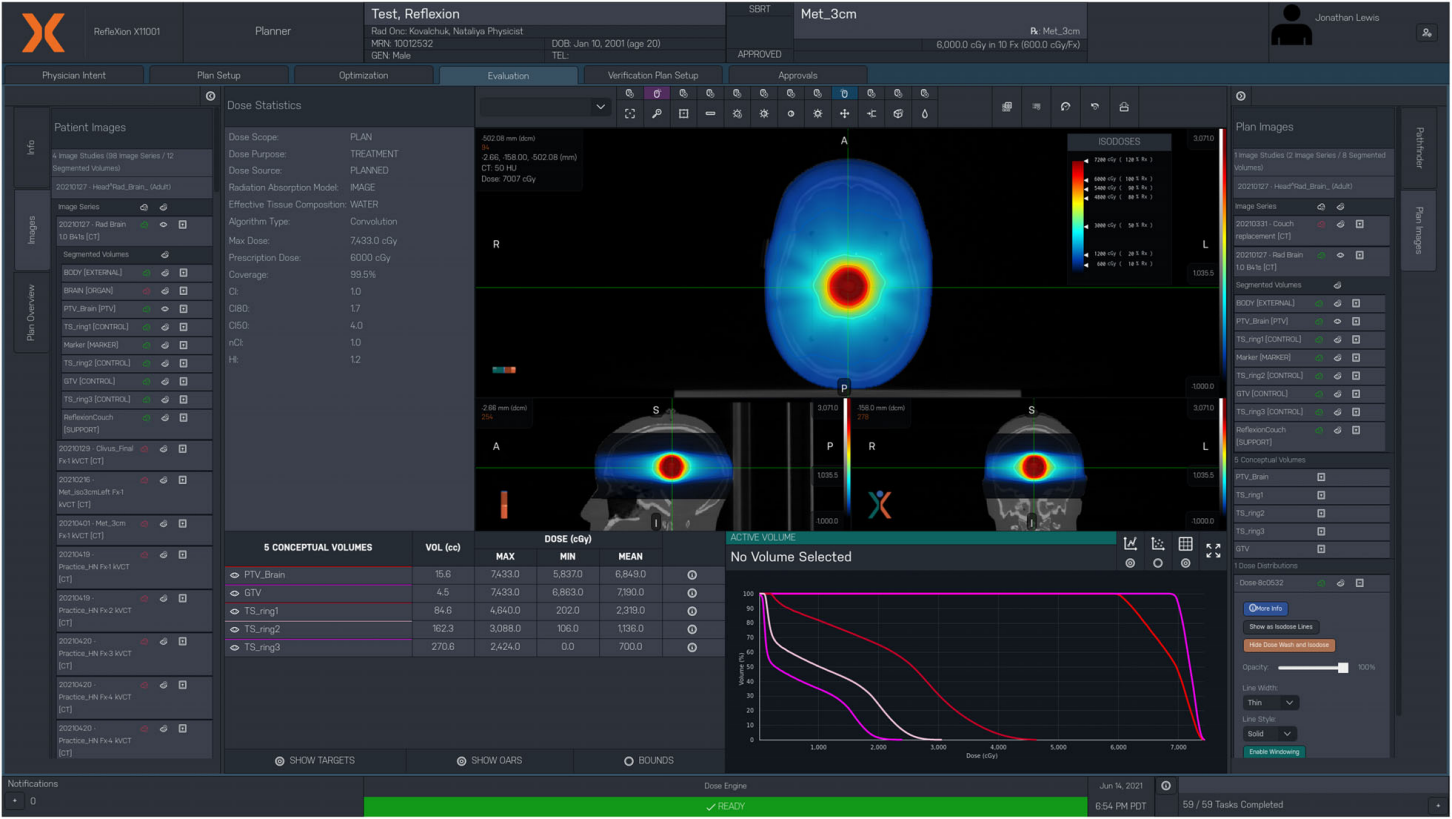
On‐axis Cyberknife head phantom treatment plan on the X1 TPS. The target for this plan was a 3‐cm‐diameter sphere in the center of the head where the prescription dose was 6000 cGy in 10 fractions. The general X1 TPS layout is also shown

A screenshot of the planned dose distribution for the on‐axis targeting test along with the general X1 TPS layout is shown in Figure 5. The total targeting error was calculated to be 0.8 mm and 1.07 mm for the on‐axis and 5 cm off‐axis plans, respectively. The Gamma passing rate (criteria of 3%/2 mm) for the 3‐cm‐diameter single brain metastasis case shifted off‐axis by 5 cm was 96.1%, indicating the X1 TPS can accurately target and deliver dose to off‐axis tumors.

**FIGURE 6:**
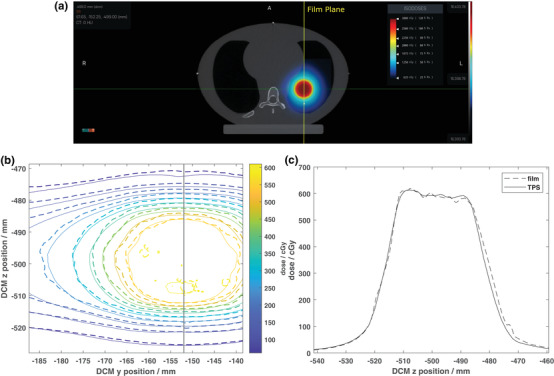
(a) Axial view of the planned dose distribution for the static motion lung SBRT plan using 1 cm jaw in the CIRS lung phantom. (b) EBT3 film‐measured (dashed lines) and TPS‐calculated (solid lines) isodose distributions for the sagittal plane through the center of the 3‐cm‐diameter target. The prescription for this plan was 2500 cGy delivered in five fractions (500 cGy per fraction). The solid black line indicates the location of a 1D profile through the dose distribution as shown in (c). Both (b) and (c) are shown in absolute DICOM coordinates where the *z*‐axis represents motion parallel to the direction of couch motion

A screenshot of the planned dose distribution for the static lung SBRT plan using 1 cm jaw is shown in Figure 6a. The measured (dashed lines) and planned (solid lines) dose distributions for the sagittal plane through the center of the target is shown in Figure 6b, and 1D profiles through the center of the target in the superior‐inferior direction are shown in Figure 6c. Excellent qualitative and quantitative (passing rate = 98.2% for criteria of 3%/2 mm) agreement between the measured and planned dose distributions can be seen in Figure 6b,c.

**FIGURE 7:**
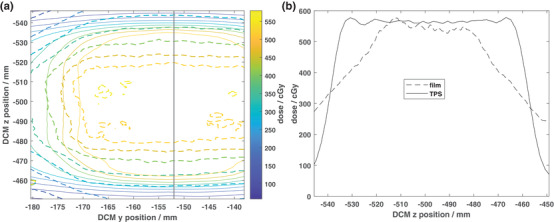
(a) EBT3 film‐measured (dashed lines) and TPS‐calculated (solid lines) isodose lines for the dynamic lung motion plan (motion amplitude = 40 mm, period = 6.0 s, motion direction = superior‐inferior) on the CIRS lung phantom. The prescription for this plan was 2500 cGy delivered in five fractions (500 cGy per fraction). The solid black line indicates the location of 1D profiles through the dose distributions as shown in (b)

The measured (dashed lines) and planned (solid lines) isodose distributions for the dynamic lung case are shown in Figure 7a, and 1D profiles through the center of the target are shown in Figure 7b. Significant under‐coverage of the target in the superior‐inferior direction can be seen in Figure 7, where the calculated 2D Gamma passing rate within 50% isodose area was 39.7% (criteria of 3%/2 mm). Repeated measurements of this treatment plan yielded similar results to those shown in Figure 7a,b.

## 4 DISCUSSION

### 4.1 Non‐dosimetric tests

With some minor exceptions, all non‐dosimetric tests passed and relevant tools were found to be functional. Such exceptions included hard‐coded CT scanners with their corresponding *HU‐to‐density curves*, missing DICOM tags in exported RT Dose files, and discrepancies between the measured and TPS‐reported collision zones in the current version of the X1 TPS. The first limitation will be corrected in subsequent releases of the X1 TPS (per communication with the manufacturer) as it limits the number of CT scanners a clinic can use to generate RefleXion plans. While the latter two items will also be corrected in software updates, an Eclipse API script, *RefleXion Assistant*, was developed in‐house to correct these deficiencies. For example, following export of the dose distribution from the X1 TPS, the user would run the *RefleXion Assistant* script in Eclipse and select the exported RT Dose file. The script would make a copy of the RT Dose file, correct the missing DICOM tag, and allow the user to import the dose file. This API script will be made freely available to the public through GitHub^@^.

Eclipse and MIM were tested in comparison to X1 as they are used to fill the gaps in the functionality of current version of X1 TPS: contouring (Eclipse/MIM), HU override (MIM), isocenter placement relative to CT reference (Eclipse), plan review/comparison to Eclipse plans (Eclipse), automated physics plan check (Eclipse), scheduling and dose tracking (Aria/Eclipse), etc. The volume differences observed in this study using TG244 head‐and‐ neck structure set were similar compared to previous study that compared contour and DVH concordance across multiple treatment planning systems.^20^, ^21^, ^22^ The largest absolute difference that was observed between Eclipse and both MIM and X1, specifically the brain contour volume (20.2 cc or 2.4%) was due to the fact that Eclipse did not extrapolate the contour to an extra half image‐slice superiorly, but MIM and X1 did. On average, the X1 DVH doses were in better agreement with the MIM DVH doses as compared to Eclipse (Table 4). The best agreement was observed for the DVH *D*_mean_ metric. Similarly to Ebert et al.^21^ and Ackerly et al.,^20^ the greatest discrepancy between the TPSs (especially Eclipse and X1) was observed in minimum doses for superficial volumes (e.g., lips, Eclipse *D*_min _= 1.8 Gy vs X1 *D*_min _= 6.9 Gy vs MIM *D*_min _= 5.2 Gy). The determination of the minimum dose is dependent not only on whether or not the TPS automatically extends volumes an extra half image‐slice for inclusion in DVH calculation, but also on the size and coincidence of the dose grid with image planes, and the interpolation methods used at the periphery of the structure.^20^, ^21^ Eclipse bins the DVH using 0.1 cGy resolution whereas RefleXion X1 uses lower resolution depending on maximum dose in the plan: 10 cGy resolution for 1000–5000 cGy *D*_max_ and 20 cGy resolution for 6000–9000 cGy *D*_max_. Increasing the resolution in the DVH binning might be advisable for the vendor to achieve in order to maintain lower volumetric and dosimetric uncertainties, especially for small SBRT/SRS target plans.^23^

The performance of the image use and display tests was in line with previously published TPS commissioning studies for other treatment planning systems.^24^, ^25^

### 4.2 Dosimetric tests

Following four iterations of the beam model, good qualitative and quantitative agreement was obtained between the measured and calculated dose profiles in water. Using the de‐ fault TPS dose grid resolution of 2.1 mm resulted in significant discrepancies between the measured and calculated crossline and inline profiles, particularly for small field sizes. A maximum mean difference in 80% of the crossline profile width of 4.1% was observed. The minimum Gamma passing rate was observed to be 55.6% over the entire profile. The maximum difference in the inline FWHM increased from 0.5 mm (1 mm dose grid) to 0.8 mm (2.1 mm dose grid). Furthermore, the TPS‐calculated profile shapes were visually different from the measured profiles when using the 2.1 mm dose grid. Similar results were observed with the output factors when the 2.1 mm dose grid was used for calculations. Upon switching to the 1 mm dose grid, the magnitude of the discrepancies between the measured and calculated profiles and output factors decreased. Currently, only the 2.1 mm grid can be used for dose calculations in the X1 TPS without manufacturer assistance. However, this will be changed in subsequent software releases to allow users to specify the dose grid resolution that should be used for calculations.

The measured and calculated doses for the two inhomogeneous solid water/bone/lung phantoms considered in this work were within 2.5%, which is within the suggested tolerance from AAPM TG 53 (3%) for central axis dose comparisons in inhomogeneous slab phantoms.^2^ The mean absolute Gamma passing rate (criteria of 3%/2 mm) for 34 cases delivered to the ArcCHECK^@^ was 97.3 ± 3.5%. Two of these plans had absolute Gamma passing rates less than 90% (the QA passing criteria at our institution). In addition, each of these failing plans was an off‐axis setup using a jaw setting of 1 cm. When the plan QA results are grouped by jaw size, the mean Gamma passing rates for the 2 cm jaw (27 plans) and 1 cm (7 plans) plans were 98.9% and 91.1%, respectively. The authors are collaborating with the ArcCHECK^@^ Sun Nuclear team to investigate these measurement discrepancies for 1 cm jaw plans and potentially introduce the ArcCHECK^@^ angular correction for RefleXion X1 plans.

With the exception of the 1‐cm‐diameter single brain metastastis case, the EBT3 film and ArcCHECK^@^ 3%/2 mm Gamma passing rates were *≥* 90% for the TG 119, single brain metastasis, and pelvis IMRT plans delivered on Solid Water (Table 5). In addition, the PinPoint‐measured and calculated doses agreed within 0.7 ± 3.1%. Greater discrepancies in the ion chamber dose comparison were observed when the chamber was placed in a high‐dose gradient region; that is, for 1.5 cm diameter brain met, the difference between measured and TPS‐calculated dose was 7.9%, but the film measurement at the treatment isocenter was within 2.1% from the calculated dose. Poor agreement was observed between the measured and calculated doses for the 1‐cm‐diameter brain metastasis case for all measurement methods. This discrepancy can be explained using the results shown in Figure 4a,b. Since the size of the target is comparable to the width of the two MLCs at the isocenter (1.25 cm), only two leaves participate in dose modulation. However, due to the limitations in the beam model, inaccuracy of tongue‐and‐groove modeling, and dose grid resolution of 2.1 mm, the X1 TPS averages the dose distribution in the target from the two leaves leading to discrepancies between the measured and calculated dose. Specifically, greater dose is measured under each MLC leaf and lower dose is measured between the leaves as compared to the TPS‐calculated dose. As this case represents the limitation in accurate TPS modeling, the smallest diameter target eligible for treatments on RefleXion X1 was set to 1.5 cm.

One of the concerns in the X1 axial step and shoot delivery with couch steps of 2.1 mm is the couch shift accuracy. As recommended by TG 148, synchronicity test was performed during machine commissioning which is designed to test the synchronization of the couch translation, gantry rotation, and accurate transmission of the beam through the MLC. The test resulted in maximum translation of 0.26 mm and maximum rotation of 0.17 degrees.^8^ The overall end‐to‐end targeting accuracy of the X1 system was calculated to be within 0.8 mm for isocentric treatment and 1.1 mm for off‐axis (5 cm shift) treatment using the Cyberknife head phantom. The isocentric test result is in line with previous findings using IGRT on TrueBeam^19^ and is within TG 135 tolerance of 0.95 mm.^26^ Furthermore, the Gamma passing rate for the 3‐cm‐diameter single brain metastasis case located 5 cm off axis was 96.1% indicating accurate delivery of the planned dose to off‐axis targets. Good agreement (Gamma passing rate = 97.1%) was observed for the static lung treatment case (Figure 6a,b). However, significant under‐coverage of the 3‐cm‐diameter target was observed for a peak‐to‐peak motion amplitude of 40 mm with a period of 6 s (Figure 7a,b).

While a peak‐to‐peak motion amplitude of 40 mm is relatively large for motion‐inclusive treatment, the current X1 system does not provide any method for respiratory gating. Due to the longer treatment times (5–15 min for conventionally fractionated treatments to 20–45 min for hypofractionated treatments)^27^ with the X1 system and limited beam hold capabilities, it is not realistic to utilize breath hold techniques for motion management. Therefore, all potential patients with target motion will be treated as motion‐inclusive for the current version of the X1 system at our institution. Per AAPM TG 76^28^ and references contained therein, observed peak‐to‐peak lung tumor motion in the superior‐inferior direction can range from 0– 50 mm depending on the size and location of the tumor. A motion amplitude of 40 mm was chosen for this work as a worst‐case scenario for treatment. However, due to the significant underdosing of the target (Figure 7), more investigation is needed to better understand the limits of the X1 system when treating moving targets with IMRT/SBRT as well as the utility of motion management systems (e.g., abdominal compression system^29^).

## 5 CONCLUSIONS

The X1 TPS commissioning results of this work agreed within the tolerances recommended by the AAPM TG reports 53, 119, 244, and 148. With some exceptions, X1 TPS was functional, including all data modification tools contained within the system. Good agreement was obtained between measured and calculated static field profiles in water, static field profiles in the presence of heterogeneities, and a range of representative clinical plans. The X1 TPS and system were able to accurately target and deliver planned dose to targets both on‐axis and off‐axis. These findings indicate the X1 TPS can, on average, accurately model dose delivery on the X1 system for modulated radiotherapy treatment plans. The results of this work have been used in the development of an ongoing QA program for the X1 TPS.

## CONFLICT OF INTEREST

Five authors of this work (T. Yeung, J. White, D. Zaks, M. Owens, S. Maganti) are employees of RefleXion Medical, Inc.

## AUTHOR CONTRIBUTIONS

E. Simiele contributed to the design of the project, data acquisition, data analysis, manuscript writing, and manuscript review and revision process. D. Capaldi contributed to the design of the project, data acquisition, data analysis, manuscript writing, and manuscript review and revision process. D. Breitkreutz contributed to the design of the project, data acquisition, data analysis, manuscript writing, and manuscript review and revision process. B. Han contributed to the design of the data acquisition methods, data acquisition, manuscript writing, and manuscript review and revision process. T. Yeung contributed to the design of the data acquisition methods, data acquisition, and manuscript review and revision process. J. White contributed to the design of the data acquisition methods, data acquisition, and manuscript review and revision process. D. Zaks contributed to modeling of the beam in the X1 TPS, interpretation of the acquired data, revising the beam model in the X1 TPS, and the manuscript review and revision process. M. Owens contributed to the modeling of the beam in the X1 TPS, interpretation of the acquired data, and the manuscript review and revision process. S. Maganti contributed to modeling of the beam in the X1 TPS, interpretation of the acquired data, revising the beam model in the X1 TPS, and the manuscript review and revision process. L. Xing contributed to the design of the data acquisition methods, data interpretation, manuscript writing, and manuscript review and revision process. M. Surucu contributed to the design of the data acquisition methods, data interpretation, manuscript writing, and manuscript review and revision process. N. Kovalchuk contributed to the design of the project, data acquisition, data analysis, manuscript writing, and manuscript review and revision process, and in addition, was responsible for overseeing the overall design of the project and clinical interpretation of the data.

## Figures and Tables

**FIGURE A1:**
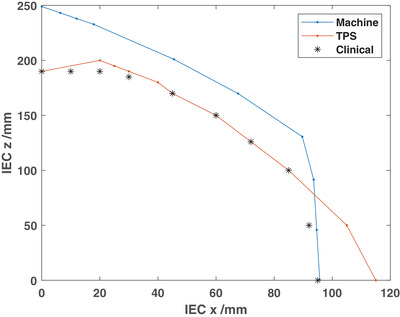
The collision zones for the X1 that were measured on the machine (labeled “Machine”), hard‐coded in the TPS (“TPS”), and the combination of the measured and hard‐coded collision zones that is used clinically to evaluate acceptable isocenter placement (“Clinical”). IEC X represents the lateral shift from isocenter and IEC Z is the couch vertical distance from couch top to treatment isocenter.
